# Analgesic Efficacy of Combinational Continuous Suprainguinal Fascia Iliaca Block and Pericapsular Nerve Group Block: A Retrospective Observational Study

**DOI:** 10.1155/anrp/9975787

**Published:** 2025-11-28

**Authors:** Kazuki Doi, Shunsuke Saima, Takashi Asai, Yasuhisa Okuda

**Affiliations:** ^1^ Department of Anesthesiology, Dokkyo Medical University Saitama Medical Center, Koshigaya, Saitama, 343-8555, Japan, saitama-med.ac.jp

**Keywords:** continuous suprainguinal fascia iliaca block, PENG block, total hip arthroplasty via anterior approach

## Abstract

**Purpose:**

We conducted a retrospective observational study to assess if the combinational continuous suprainguinal fascia iliaca block and pericapsular nerve group block would be effective in inhibiting postoperative pain in patients undergoing total hip arthroplasty.

**Methods:**

In 40 patients who underwent elective total hip arthroplasty via anterior approach (April, 2023–April, 2024), suprainguinal fascia iliaca block and PENG block were performed with 25 mL of 0.2% levobupivacaine before induction of general anesthesia. Postoperatively, 0.125% levobupivacaine was continuously infused (at 4 mL h^−1^) for suprainguinal fascia iliaca block. Postoperatively, the numerical rating scale (NRS) of pain at rest was recorded immediately after surgery and at 2, 6, 12, 24, and 48 h after surgery. Primary outcome measure was the incidence of rebound pain, and secondary outcome measure was the incidence of postoperative acute pain.

**Results:**

Postoperative acute pain was observed in 22 of 39 patients (56% [95% confidence interval: 40%–72%]). In the remaining 17 patients, rebound pain was observed in 2 (12% [95% confidence interval: 0%–27%]).

**Conclusions:**

We conclude that in the patients who underwent total hip arthroplasty via anterior approach, combinational use of continuous suprainguinal fascia iliaca block and PENG block may frequently be insufficient to prevent postoperative acute pain but may be effective in reducing the incidence of rebound pain.

## 1. Introduction

For postoperative analgesia after total hip arthroplasty, the guideline for total hip arthroplasty (PROSPECTguideline [1]) recommends suprainguinal fascia iliaca block.

Pericapsular nerve group block (PENG block) has been reported to be effective in reducing pain after hip fracture and total hip arthroplasty [2–4] and is less likely to cause motor nerve block [5]. Nevertheless, PENG block does not provide sufficient analgesia to skin incision and subcutaneous dissections during total hip arthroplasty because the area is typically located on the lateral thigh, which is supplied by the lateral femoral cutaneous nerve [3, 6, 7]. A study of combinational use of suprainguinal fascia iliaca block and PENG block in patients undergoing hip surgery [6] indicated that single‐shot block could effectively prevent postoperative acute pain.

One major problem with peripheral nerve block is postoperative rebound pain [8]. Rebound pain is usually defined as an acute increase in pain intensity after analgesic effect of a peripheral nerve block has receded, typically manifesting within 24 h after the block [9]. In addition, the incidence of rebound pain is higher in patients who received single‐shot peripheral nerve block and general anesthesia than in those received general anesthesia only [10, 11].

We considered that a continuous infusion of a local anesthetic to the block site would be required to reduce postoperative acute pain and rebound pain. Although continuous peripheral nerve block has been suggested to prevent postoperative rebound pain [12, 13], there have been no reports which assessed if continuous suprainguinal fascia iliaca block effectively inhibits postoperative acute pain and rebound pain, in patients who underwent total hip arthroplasty.

We conducted a retrospective observational study to assess if the combinational continuous suprainguinal fascia iliaca block and PENG block would be effective in inhibiting postoperative acute pain but and rebound pain, in patients undergoing total hip arthroplasty via anterior approach.

## 2. Methods

The research ethics committee of Dokkyo Medical University Saitama Medical Center approved the study (ID: 24008), and written informed consent was obtained from all the patients.

**Figure 1:**
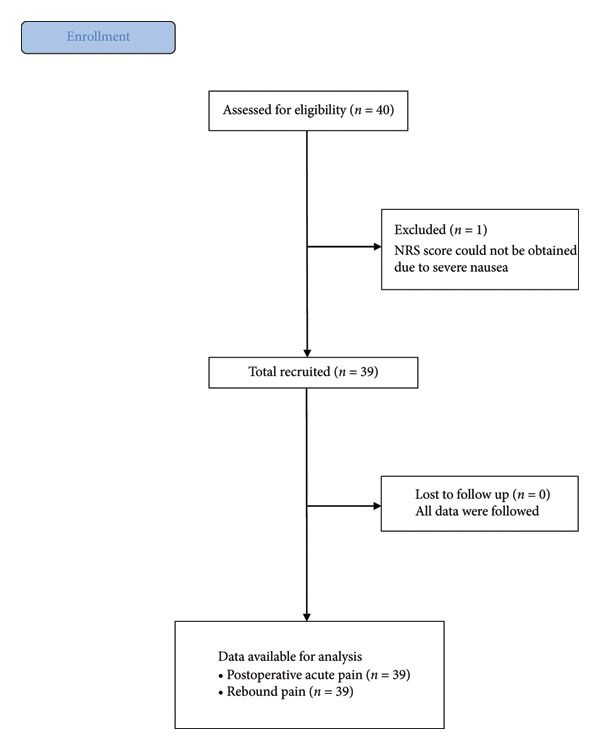
STROBE diagram.

As a retrospective observational study, we studied 40 patients (age ≥ 20 years) who underwent elective total hip arthroplasty via anterior approach (but not posterior approach) (April, 2023–April, 2024). Patients with American Society of Anesthesiologists’ (ASA) physical status classification system 3 or greater, morbidly obese (BMI ≥ 35 kg m^−2^), and allergic to local anesthetics were excluded from this study (Figure 1).

The anesthesia method of patients was standardized as follows: In the operating room, routine monitors, such as a blood pressure cuff and an electrocardiogram, were applied, and an intravenous infusion was started. Fentanyl 50 μg was injected intravenously.

After local infiltration of 1% lidocaine to the puncture site, PENG block was performed using a 20‐gauge 100‐mm needle (Stimuplex Ultra 360, B Braun, Tokyo, Japan), under ultrasound‐guide in‐plane technic using a C 1–5‐MHz convex probe (PX, Fujifilm Sonosite, Tokyo, Japan). After negative aspiration of blood, 25 mL of 0.2% levobupivacaine was injected in the musculofascial plane, between the psoas tendon and the pubic ramus. Infusion of the local anesthetic to the correct position was confirmed ultrasonographically, by observing formation of a hydro dissection and spread under the iliopsoas muscle.

Suprainguinal fascia iliaca block was then performed by out‐of plane technic using an 18‐gauge 100‐mm needle (Contiplex Tuohy Ultra360, B Braun, Tokyo, Japan), under the guidance of an ultrasonograph A4 15‐MHz linear probe (PX, Fujifilm Sonosite, Tokyo, Japan). After negative aspiration of blood, 25 mL of 0.2% levobupivacaine was injected underneath the fascia iliaca toward the suprainguinal area, and the correct injection was confirmed by observing the separation of the iliacus muscle by hydro dissection on the ultrasound monitor.

A 20‐gauge 1,000‐mm catheter (Contiplex Tuohy Ultra360, B Braun, Tokyo, Japan) was then inserted through the needle to the suprainguinal area, approximately 4 cm beyond the tip of the needle. The needle was removed, and the catheter was fixed to the skin using a drape with a transparent window in the center (Tegaderm, I. V. Advanced, 3M Health Care, Neuss, Germany). Over the drape, another larger drape with a cover film (PERME‐ROLL, Nitto, Tokyo, Japan) was attached over the initial drape. This double draping allowed surgeons to disinfect the surgical field and to remove the outer drape after completion of surgery.

A facemask was placed over the patients’ mouth and nose, and 6 L min^−1^ oxygen was provided for at least 3 minutes. General anesthesia was then induced with propofol 2 mg kg^−1^ and fentanyl 2 μg kg^−1^, and neuromuscular blockade was achieved with rocuronium 0.6 mg kg^−1^. When neuromuscular blockade was judged adequate, the trachea was intubated. Anesthesia was maintained with sevoflurane in oxygen and continuous infusion of remifentanil 0.1–0.2 μg kg^−1^ min^−1^. Total hip arthroplasty via anterior approach was carried out, and after the surgery, the patient was awaken from anesthesia. Postoperatively, 0.125% levobupivacaine was continuously infused with the rate of 4 mL h^−1^, and 1,000 mg of paracetamol was administered intravenously. Infusion of levobupivacaine was continued for approximately 2 days after surgery, and paracetamol was prescribed at intervals of every 6 h for approximately 5 days after surgery.

Postoperatively, the numerical rating scale (NRS) of pain at rest was recorded immediately after surgery by an anesthesiologist in charge and at 2, 6, 12, 24, and 48 h after surgery by ward nurses. The analgesic effect of the block on postoperative acute pain was judged effective when NRS was 3 or lower, during the initial 6 h after surgery. Rebound pain was defined as the transition from well‐controlled pain NRS 3 or lower during the initial 6 h to severe pain NRS 7 or greater within 24 h of a peripheral nerve block [9].

Primary outcome measure was the incidence of rebound pain, and secondary outcome measure was the incidence of postoperative acute pain. 95% confidence interval for the incidences was calculated.

## 3. Results

Patients’ characteristics are shown in Table 1. In all the patients, surgery was carried out successfully without being interfered with the presence of the drapes over the catheter, and the patient was awaken from anesthesia.

**Table 1:** Patients’ characteristics.

Age, mean (SD)	65 (12.1)
Male/female (n)	7/33
Height, cm mean (SD)	157 (7.9)
Weight, kg mean (SD)	60 (12.3)
BMI; kg/m^2^ mean (SD)	24 (4.7)

Rebound pain was observed in 2 of 17 (12% [95% confidence interval: 0%–27%]). Postoperative acute pain was observed in 22 of 39 patients (56% [95% confidence interval: 40%–72%]). No complications which required treatment occurred in any patients.

## 4. Discussion

We have found that although the incidence of postoperative acute pain was still high (56%) even after combinational use of continuous suprainguinal fascia iliaca block and PENG block, continuous infusion was likely to reduce the incidence of rebound pain.

In the past, we were performing suprainguinal fascia iliaca block, with or without PENG block, but had founded acute pain frequently occurred postoperatively. This was the reason for introducing continuous infusion of a local anesthetic to the block site. Nevertheless, it became apparent that the incidence of postoperative acute pain was still high.

Our result was different from the previous report [6] of 10 patients, in whom single‐shot block could effectively prevent postoperative acute pain. The reason for this discrepancy is not clear. One possibility is that, in our patients with postoperative acute pain, local anesthetic could not have spread sufficiently to the suprainguinal area. Nevertheless, this is unlikely, because ultrasonography confirmed formation of a hydro dissection in the musculofascial plane between the psoas tendon and the pubic ramus. Second possibility is that we used only paracetamol except for peripheral nerve block and did not use patient controlled intravenous analgesia with opioids or nonsteroidal anti‐inflammatory drugs. Our study has confirmed that, even when a continuous supranguinal fascia iliaca block is being used, multimodal analgesia may frequently be requested to minimize acute pain [14].

The incidence of rebound pain after a single‐shot peripheral nerve block for ambulatory upper and lower limbs surgery (without general anesthesia) has been reported to be 50% [9]. In contrast, in our study, rebound pain occurred in only 2 of 17 (12%) of patients. Therefore, it is likely that continuous peripheral nerve block may be effective in reducing the incidence of rebound pain.

Limitations of this study include that this is a retrospective observational study, so that the true efficacy is not clear of continuous suprainguinal fascia iliaca block in inhibitory effect on postoperative acute pain and on rebound pain.

In conclusion, in patients who underwent total hip arthroplasty via anterior approach, combinational use of suprainguinal fascia iliaca block and PENG block, may frequently be insufficient to prevent postoperative acute pain but may be effective in reducing the incidence of rebound pain.

## Conflicts of Interest

The authors declare no conflicts of interest.

## Funding

This research received no external funding.

## Data Availability

The data that support the findings of this study are available on request from the corresponding author.
